# The 2022 Massive Open Online Course (MOOC) to train physiotherapists in the management of people with spinal cord injuries: a qualitative and quantitative analysis of learners’ experiences and its impact

**DOI:** 10.1038/s41393-023-00922-1

**Published:** 2023-08-14

**Authors:** Joanne V. Glinsky, Jocemar Ilha, Yuan Xiong, Guillermo Gomez, Stephan Rostagnor, Soraya Martín-Manjarrés, Keira Tranter, Stephen Muldoon, Eric Weerts, Lisa A. Harvey

**Affiliations:** 1 Kolling Institute, Faculty of Medicine and Health, The University of Sydney, Sydney, NSW Australia; 2 John Walsh Centre for Rehabilitation Research, Northern Sydney Local Health District, St Leonards, Sydney, NSW Australia; 3 Physiotherapy Department, Universidade do Estado de Santa Catarina (UDESC), Florianópolis, Brazil; 4 Guangdong Work Injury Rehabilitation Hospital, Guangzhou, Guangdong China; 5 Kreativa Rehabilitación, Buenos Aires, Argentina; 6 Aix-Marseille University, Marseille, France; 7 National Hospital for Paraplegics in Toledo, Toledo, Spain; 8 International Spinal Cord Society, Aylesbury, UK; 9 Humanity & Inclusion, Brussels, Belgium

**Keywords:** Health care, Neurology

## Abstract

**Study design:**

Observational.

**Objectives:**

To determine the learners’ experience and the impact of a Massive Open Online Course (MOOC) conducted to teach physiotherapists about the management of people with spinal cord injuries (SCI).

**Methods:**

A SCI MOOC for physiotherapists was run in 5 different languages at the end of 2022. Qualitative and quantitative data were collected from different sources including registration details, pre- and post-MOOC Knowledge Assessments, a post-MOOC Evaluation, social media posts and online tracking of websites and emails. The data were used to answer four key questions: (i) what was the reach of the MOOC, (ii) what did participants think about the MOOC (iii) did the MOOC change participants’ knowledge and/or confidence, and (iv) did the MOOC change participants’ clinical practice or the way they teach others?

**Results:**

25,737 people from 169 countries registered for the MOOC. 98% of participants who completed the Evaluation (*n* = 2281) rated the MOOC as either “*good*” or “*very good*”. Participants’ knowledge improved by a median (IQR) of 25% (10 to 45%) (*n* = 4016 participants) on the MOOC Knowledge Assessment. Participants reported changes in confidence, and intentions to change clinical practice and incorporate what they had learnt into the way they teach others in response to the MOOC.

**Conclusion:**

The MOOC provided an efficient way to increase physiotherapists’ knowledge about the physiotherapy management of people with SCI. Participants enjoyed the MOOC, and indicated an intention to change clinical practice and the way they taught others.

## Introduction

Physiotherapists often receive little education or training on the physiotherapy management of people with spinal cord injuries (SCI) during their undergraduate or post-graduate courses. This is because often universities (i) do not prioritise this topic given the relatively low incidence of SCI, and (ii) do not have staff with the expertise and/or confidence to teach students about SCI. This lack of training in SCI can compromise the quality of physiotherapy provided to people with SCI. There is, therefore, a need to upskill physiotherapy students as well as physiotherapists new to SCI, and teach them about the physiotherapy management of people with SCI. To address this need, we designed a 5-week Massive Open Online Course (MOOC) in collaboration with the International Spinal Cord Society. This was run in 2014 [1–3], 2016, 2018 [4] and 2022. However, this paper is only about the most recent MOOC run in Nov/Dec 2022. The 2022 MOOC had 2.5 times as many participants as the 2018 MOOC and was run in 5 languages (all previous MOOCs have only been run in English).

Massive Open Online Courses are free online courses characteristed by a curriculum and large numbers of participants [5–7]. They are an increasingly common and popular way of providing training and education without the need for costly face-to-face teaching [5, 8–16]. MOOCs are also a part of a social movement driven by a communal belief in the importance of free high-quality education for all [17, 18]. Different types of MOOCs on an array of topics are provided through organisations such as edX, Coursera, FutureLearn, Audacity, Udacity and The Khan Academy [16–20]. They all come in many different formats and utilise various teaching strategies [16]. Some just consist of recorded online video lectures [18] whilst others require students to move through the equivalent of an online textbook. Most include a mix of online discussions, links to further readings and opportunities for self assessment.

Recent work has been directed at trying to better evaluate the structure and success of MOOCs. Many believe that MOOCs need to develop an online community so that participants feel as though they are learning with others [8, 11, 17, 21]. This may increase enjoyment and a mutual and widespread commitment to the completion of a MOOC. A notable study [21] reviewed 33 medical MOOCs and assessed each on 11 key features considered important for adult learning including whether they created an online community. These included features such as: (i) providing learners with different ways to learn to cater for different needs and learning styles; (ii) providing content and problems that draws from the real world (in our case, real physiotherapists and people with SCI from a variety of countries); (iii) building on existing knowledge and then presenting new knowledge that is then applied to real clinical scenarios; (iv) giving learners the opportunity to apply new knowledge; (v) giving learners the opportunity to interact and learn with others; and more (see [21] Table 1, pg 157). Our MOOC incorporated many of these features although future work could (and should) be directed at objectively assessing our MOOC on each of these 11 key principles with the aim of further developing and improving what we currently provide.

**Table 1:** REACH: the number of registrants from the 10 countries with the most participants.

Egypt	7255 (28%)
China	2782 (11%)
India	2580 (10%)
Australia	1393 (5%)
Brazil	1009 (4%)
United Kingdom	919 (4%)
Saudi Arabia	655 (3%)
Nigeria	630 (2%)
Pakistan	568 (2%)
France	424 (2%)

% reflects the percentage of all registered participants.

MOOCs are not without their critics [17]. Some dislike the teaching style of MOOCs. Others argue that they can not be used to teach clinical or practical skills (clearly important for physiotherapists). And most acknowledge that many who sign up to MOOCs never complete them with some authors reporting completion rates as low as 4% [7, 19, 20]. Poor completion rates are a concern but many argue that completion rates are not a good indication of the success of a MOOC. Instead the success should be measured with respect to what each learner wants to achieve from a MOOC [6, 7, 21]. This will vary from individual to individual and depends on people’s access to other learning opportunities. Some participants may be content to merely dabble in parts of a MOOC that are directly relevant to them [7, 16]. Irrespective, the very presence of a MOOC on a particular topic (such as the physiotherapy management of people with SCI) may help flag the importance of the topic, which may in turn help prioritise other educational initiatives in the area. It may also direct large numbers of people to available resources.

A better understanding of the impact of MOOCs is important for making decisions about the value of running future MOOCs. In addition, it is important to know whether they are effective and whether learners value them. This type of information can be used to improve future MOOCs. The aim, therefore, of this study was to determine the learners’ experience and the impact of our 2022 MOOC conducted to teach physiotherapists about the management of people with SCI. To address these two aims, we posed four questions based on the RE-AIM and Kirkpatrick frameworks as used by others to evaluate the success of training courses and MOOCs [16, 18, 22, 23]. The questions were:

1. THE REACH: Who were the participants and were they engaged?
2. THE EFFECTIVENESS: Was there a short-term/immediate change in knowledge and/or confidence?
3. REACTION: How did the participants react to the MOOC?
4. BEHAVIOUR: Will the participants change their clinical practice or teach others differently in response to the MOOC?

## Methods

### Background to the MOOC

The MOOC was 5 weeks in duration and ran between 7th November to 11th December 2022. It was free and provided in five different languages (English, Spanish, French, Portuguese and Chinese). It was intended for physiotherapy students or junior physiotherapists with little prior experience in the area of SCI. Participants were required to devote 5 h per week for 5 weeks to the MOOC (a total of 25 h). They were required to complete online lessons, engage with additional resources and contribute to an online discussion forum. They could do these activities at any time during the week. That is, participants were not required to be online together at particular times. Participants were also given access to a textbook that the MOOC was based upon [24]. Participants were given certificates of completion if they sat the final post-MOOC Knowledge Assessment. The MOOC was run in collaboration with the International Spinal Cord Society and based on similar MOOCs run by some of the authors in 2014, 2016 and 2018 [1, 2].

The MOOC was housed on a purpose-built website—www.SCIMOOC.org. This website provided all the objectives of the MOOC as well as the objectives for each lesson (see Supplementary File 1). It also outlined tasks for each week. Some of the tasks required participants to move across to physiotherapy-specific online lessons housed at www.elearnSCI.org (created by ISCoS). The lessons contain over 1500 screens and 150 videos. Each screen has a small amount of text with an accompanying image or video. Interspersed are screens with activities that require participants to answer questions or identify appropriate physiotherapy exercises for a particular problem. The screens were designed to encourage participants to repeatedly *stop, think, do [something] and revise [what they had learnt]*. The content was largely built around real-world case studies with videos of people with SCI and experienced SCI physiotherapists from countries around the world. Participants were frequently presented with different clinical problems and prompted to reflect on appropriate assessments and treatments. The elearn website also contains multiple-choice assessments at the end of each lesson that provide participants with an opportunity to test their knowledge.

Participants were encouraged to join and engage with their colleagues and course co-ordinators (teachers) on a closed Facebook (FB) group. There was one FB group for each of the four languages (English, French, Spanish and Portuguese) and a Weibo chat forum for Chinese participants (because FB is blocked in China). Each FB group had one or two language co-ordinators who oversaw the FB group and responded to participants’ posts. Two or three discussion threads were opened by the coordinators each week. These posed particular clinical scenarios or questions that related to the week’s learning content. Participants were encouraged to post to these open threads but they could not open new discussion threads of their own. The discussion threads were closed at the end of each week.

Participants were instructured on what to do each week through email. The instructions for each week were also posted on the FB groups and on the MOOC website. In addition, short videos were created in English and Portuguese at the beginning of each week for participants to view. These outlined the content for the week. These videos were not created in the other languages because of financial constraints.

The overall MOOC co-ordinators and the language coordinators were all senior physiotherapists with clinical experience in the area of SCI. Three of the co-ordinators held academic positions and regularly taught physiotherapy students and junior physiotherapists about the management of SCI. Each co-ordinator made a short video to introduce themselves to participants.

#### Assessing participant’s knowledge and seeking their feedback

Participants were asked to complete a pre- and post-MOOC Knowledge Assessment, and a post-MOOC Evaluation. The Knowledge Assessments consisted of 20 multiple choice questions. Prior to the commencement of the MOOC pairs of questions were prepared and then one question of each pair was randomly assigned to the pre-MOOC Knowledge Assessment and the other to the post-MOOC Knowledge Assessment. This was done to ensure that there were no systematic differences in the difficulty of the questions comprising the pre- and post-MOOC Knowledge Assessments. The score attained on the first attempt of the post-MOOC Knowledge Assessment was printed on the participants’ certificates of completion. The post-MOOC Evaluation asked participants to rate statements about the MOOC on a five-point scale ranging from “*strongly disagree*” to “*strongly agree*”. There were also open-ended questions in which participants could provide free text feedback.

The University of Sydney’s Ethics Committee approved this study and provided a waiver of consent on the basis that the study met the criterion for waiver of consent as articulated in The National Statement on Ethical Conduct in Human Research (pg 21) [25].

### Data collection

Qualitative and quantitative data were collected to address the four posed questions captured by the themes: reach, effectiveness, reaction and behaviour. The data sources were participants’ registration details, the pre- and post-MOOC Knowledge Assessments, the post-MOOC Evaluation and the FB posts. In addition, online tracking was used to determine the number of hits or views of the websites, videos and emails that were part of the MOOC. The details are outlined below.

#### THE REACH: Who were the participants and were they engaged?

##### The participants

Data were collected for each registered participant for each of the five languages in which the MOOC was run including their country and level of SCI experience (undergraduate student or postgraduate student or <1 year SCI experience or 2 to 5 years SCI experience of >5 years SCI experience).

##### Engagement

This was determined by looking at the number of people who accessed, completed or viewed different components of the MOOC including:

Pre- and post-MOOC Knowledge Assessments and the post-MOOC Evaluation: The number of participants who completed the pre- and post MOOC Knowledge Assessments and the post-MOOC Evaluation were counted (data sourced from the Content Management System of the www.SCIMOOC.org website).

Facebook or Weibo Group: The number of participants who joined each FB or Weibo Group, and posted comments or reacted to posts were counted (data sourced from FB or Weibo).

Emails: The number of instructional emails that were sent and opened were counted (data sourced from MailChimp).

Website hits on www.SCIMOOC.org: The number of page views of www.SCIMOOC.org were recorded (data sourced from Google analytics). This website contained the weekly instructions.

Website hits on www.elearnSCI.org: The number of page views of www.elearnSCI.org were recorded (data sourced from by Google analytics). This website contained most of the learning content.

Daily views of www.physiotherapyexercises.com: The number of page views of www.physiotherapyexercises.com were recorded over the duration of the MOOC although participants were only asked to use this website during the 4th week of the course to create exercise booklets for a patient (data sourced from Google analytics).

##### Attrition rate

This was estimated on the basis of the number of participants who completed the post-MOOC Knowledge assessment. It was also based on the change in the number of page views of www.SCIMOOC.org and the www.elearnSCI.org websites from the beginning to the end of the MOOC, and the drop in the number of comments posted to the threads each week on FB.

#### THE EFFECTIVENESS: Was there a short-term/immediate change in knowledge and/or confidence?

This was determined in the following ways:

##### Knowledge assessments

The median (interquartile range, IQR) change in scores from the pre-MOOC and post-MOOC Knowledge Assessments was determined in the sub-sample of participants who completed both Assessments.

##### Text analysis

The comments posted to three discussion threads on the FB Pages and the text responses received on the English version of the post-MOOC Evaluation were searched for the following terms—“*learnt*”, “*learned*”, “*learn*”, “*did not know*” and “*understand*” and “confidence”. Each mention was counted to gauge participants’ self-reported changes in knowledge and confidence.

#### REACTION: How did the participants react to the MOOC?

##### Post-MOOC evaluation

The results of the post-MOOC Evaluation were used to gauge how participants reacted to the MOOC.

##### Text analysis

The comments posted to three discussion threads on the FB Groups, and the text responses received on the English version of the post-MOOC Evaluation were used to determine:

Participants’ overall impressions of the MOOC: The number of times positive words such as “*amazing*”, “*awesome*”, “*brilliant*”, “*fantastic*”, “*great*”, “*superb*” or “*wonderful*” were used to describe the MOOC were counted.

The topics that participants enjoyed or valued learning about: The FB threads and post-MOOC Evaluation asked participants to reflect on what they had learnt or enjoyed learning about. The leading 8 topics participants wrote about were identified and counted.

The aspects of the learning experience that participants valued and/or enjoyed: The number of times participants mentioned different aspects of the learning experience in the FB threads and post-MOOC Evaluation were identified and counted.

#### BEHAVIOUR: Will the participants change their clinical practice or teach others differently in response to the MOOC?

##### Text analysis

The comments posted in the FB threads and post-MOOC Evaluation were used to determine participants’ intention to change clinical practice in response to the MOOC and to teach others differently.

## Results

### THE REACH: Who were the participants and were they engaged?

#### The participants

**Fig. 1:**
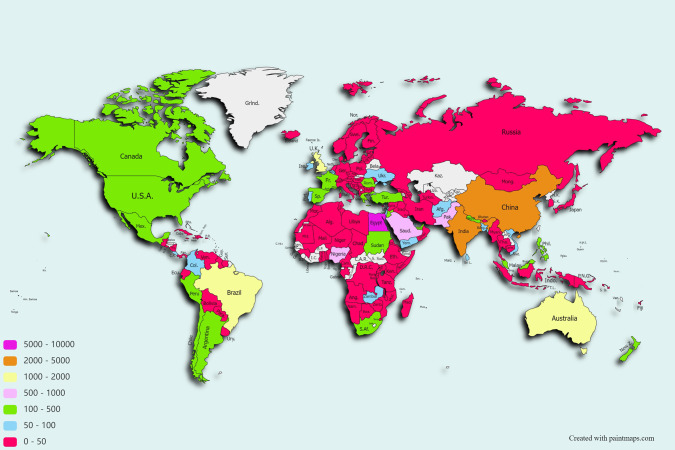
REACH: World map indicating the country of origin of the participants.

25,737 participants registered for the MOOC (see Supplementary File 2). Participants were from 169 countries (see Fig. 1 and Supplementary File 3) and had a mix of experiences with SCI (see Supplementary File 4). 48% were either undergraduate or post-graduate students. The countries with the most registrants were: Egypt, India, China, Australia and Brazil (see Fig. 1 and Table 1).

#### Engagement

The results for each measure of engagement are as follows:

Pre- and post-MOOC Knowledge Assessments and the post-MOOC Evaluation: 10,206 and 4873 participants completed the pre- and post-MOOC Knowledge Assessments, respectively, and 2281 participants completed the post-MOOC Evaluation (see Supplementary File 5).

**Fig. 2:**
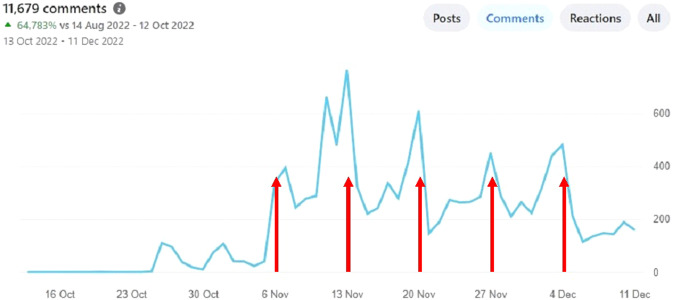
REACH: the number of comments on the English version of the Facebook Page over the duration of the MOOC. The arrows indicate the beginning of weeks 1, 2, 3, 4, and 5.

Facebook or Weibo Group: 16,298 participants joined one of the FB or Weibo Groups, and 13,725 participants of the FB groups were classified by FB as “*active members*” (the equivalent figures were not provided by Weibo) (see Fig. 2, Supplementary File 6 and Supplementary File 7). There were approximately 20,000 comments posted and 22,000 reactions to comments (see Supplementary File 6). The number of comments to each discussion thread for the English FB Group started at 1400 in the first week and decreased to 650 by the last week (see Supplementary File 6). This was equivalent to between 200 and 700 posts per day (see Fig. 2).

Emails: The number of emails sent ranged from 14,000 (2 weeks prior to the MOOC) to 25,271 (at week 5) (see Supplementary File 8). Nearly all were received. The emails sent in the first week with the initial instructions were opened by all (some multiple times) but only opened by 40% of participants by week 5 (the weekly instructions were also posted on the MOOC website and the FB pages, so participants may have opted to read the instructions for each week here rather than on their emails as time progressed).

**Fig. 3:**
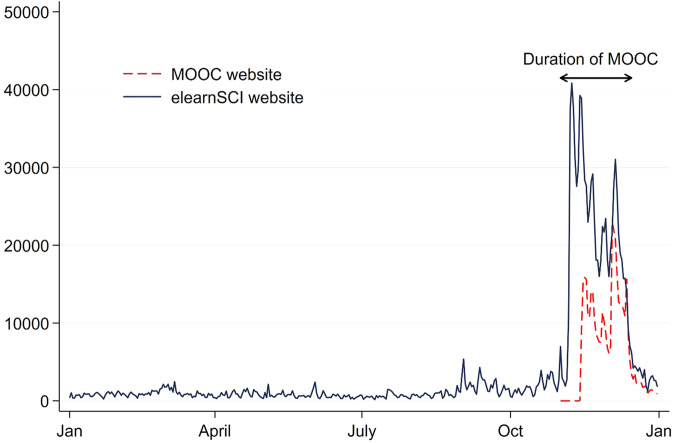
REACH: Usage of www.SCIMOOC.org and www.elearnSCI.org. Number of views per day of the www.SCIMOOC.org and www.elearnSCI.org websites over 2022. The duration of the MOOC is indicated. The median (IQR) number of page views per day of www.elearnSCI.org in the 6 months prior to the MOOC was 649 (464 to 911) and in the 5 weeks of the MOOC was 23,150 (18,091 to 29,325). There was a 33% and 7% drop off in the number of page views from the weeks with the highest number of page views per day to the weeks with the lowest number of page views per day for the www.SCIMOOC.org and www.elearnSCI.org websites, respectively. Capture of the Google Analytics data for www.MOOC.org commenced 5 days after the start of the MOOC (and may therefore be an underestimation of page views).

Website hits on www.SCIMOOC.org: There was a median (IQR) of 10,712 page views per day (6872 to 14,050) (see Fig. 3)

Website hits on www.elearnSCI.org: There was a median (IQR) of 23,150 page views per day during the 5 weeks of the MOOC (18,091 to 29,325) (see Fig. 3). This was equivalent to a 35 time increase in usage compared to pre-MOOC (figures taken from the 6 months prior to the MOOC). There was a decrease by 7% from week 1 when page views were the highest (14,537) to week 5 when page views were the lowest (13,583).

Daily views of www.physiotherapyexercises.com: Page views increased from 10,000 per day (prior to the MOOC) to 16,000 per day over week 4 and week 5 of the MOOC (see Supplementary File 11) when participants were required to use this website for an activity in week 4.

#### Attrition rate

The measures of attrition varied from as low as 7% to as high as 81%. For example, only 19% of participants completed the post-MOOC Knowledge Assessment (indicative of a dropout rate of 81%). And 60% of those who engaged with FB initially stopped engaging by 5 weeks (see Fig. 2, Supplementary Files 6 and 7). However, there was only a 7% decrease in the page views per day of the www.elearnSCI.org website (see Fig. 3) over the 5 weeks. The decrease in the usage of www.SCIMOOC.org was higher at a 33% decrease in the page views per day (see Supplementary Files 9 and 10). Taken together, and assuming the page views of www.elearnSCI.org provide the most robust indication of engagement, our best estimate is that approximately 25% of those who commenced the MOOC moved through most of the learning content and remained engaged.

### THE EFFECTIVENESS: Was there a short-term/immediate change in knowledge and/or confidence?

#### Knowledge assessments

10,206 participants sat the pre-MOOC Knowledge Assessment, 4873 participants sat the post-MOOC Knowledge Assessment and 4016 participants sat both the pre- and post-MOOC Knowledge Assessments. Of those who sat both the pre- and post-MOOC Knowledge Assessments, the median scores increased from 55% (40 to 65) to 90% (71 to 100) with a median (IQR) change in scores of 25% (10 to 45) (see Supplementary File 12).

#### Text analysis

There were 1639 comments made by participants in the three discussion threads on the FB Pages and the post-MOOC Evaluation that mentioned the words – “*learnt*”, “*learned*”, “*learn*”, “*did not know*” and “*understand*” (see Table 2 for some examples of the comments). There were 64 comments made by participants that mentioned the words – “*confidence*” (see Supplementary File 13 for some examples of the comments).

**Table 2:** Examples of comments on Facebook and the post-MOOC Evaluation indicative of (a) Effectiveness: a change in knowledge; (b) Behaviour: an intention to change clinical practice; and (c) Reaction: participants’ satisfaction with the MOOC.

Source	(a) EFFECTIVENESS: A change in knowledge
FB, Wk 2	I always confuse while using ICF but *learned* it well now
FB, Wk 2	I *did not know* that there were so many outcome measures for SCI.
FB, Wk 2	I have *learnt* that for proper and effective way to manage patients with SCI
FB, Wk 2	I have *learnt* about ASIA assessment form ..As i was always confuse about this
FB, Wk 2	I really *learnt* many thing related spinal injury and its classification and its effects on the other parts of the body
FB, Wk 2	….*learning* in more detail about the complexities has been intriguing.
FB, Wk 2	I have *learned* a lot about the wheelchair mobility training.
FB, Wk 4	this week I have *learned* more in depth the subtasks that are required to perform fine motor skills.
FB, Wk 4	I *learned* to give simpler guidelines and with a simple language so the patient understands better
FB, Wk 5	I’ve *learned* a lot from the course
FB, Wk 5	I enjoyed *learning* to make an exercise program for SCI patients using the physiotherapyexercises.com website.
FB, Wk 5	I have *learned* a lot from this course
Evaluation	I’m a visual learner so the videos and photos *help me understand* and enhance my observation skills as a clinician.
Evaluation	Testing throughout ensured you took the information in and allowed you *learn* from things you thought you understood but didn’t (if you got it wrong).
Evaluation	I liked the self-assessments and discussion boards on facebook - *learned* a lot from my peers.
Evaluation	I have *learnt* so much. All the information was in one place, it was thorough and concise.

	(b) BEHAVIOUR: An intention to change clinical practice
FB, Wk 4	I learned *how teach the patients proper* transfer
FB, Wk 4	The most important thing I learned actually is *the use of physiotherapyexercises.com*
FB, Wk 4	I have learnt the importance of continuous repetitive practice in teaching patients motor skills
FB, Wk 4	I have learnt *how to be good teacher* when I learn patient new task to do by give a simple and clear instruction, give feedback about result and performance
FB, Wk 4	This week I gained a lot of new ideas and teaching tools for *breaking down large motor movements into sub tasks*
FB, Wk 4	I learned how *breaking up a functional task into smaller sub-tasks* can be a very systematic way of training or retraining a patient, it is a very organised way of providing therapy,
Evaluation	Some of what I learned in the course was immediately *put to use in my actual clinical practice*
Evaluation	*I will use* the knowledge and skills to further improve myself
Evaluation	Brilliant professional development and I will definitely be able *to put in practice* the knowledge and skills learnt
Evaluation	I *will improve myself* with the acquired knowledge in my clinical practice
Evaluation	This course help me alot. *it improve my skills*
Evaluation	*I already have improved my practice* and have used bit and pieces that i have learned in work.
Evaluation	*I will definitely use what I have learned to improve myself*

	(c) REACTION: Satisfaction with the MOOC^a^
FB, Wk 4	Thank you so much for creating this awesome online course and giving each and every one of us the chance to learn. It’s the most complete and systematic online course i ever have done
FB, Wk 5	Hats off to the coordinators of this course to bring all information and resources for SCI under one course.
FB, Wk 5	I really learn alot this course even change my thinking approach and treatment strategies of my pts with SCI. Actually I don’t have enough words to thank you.
FB, Wk 5	This course was more than an eye opener for me.
FB, Wk 5	This course was brilliant. It will enhance my practice and no doubt improve experience of service users.
FB, Wk 5	This course is just awesome. The way you have designed it content pattern to make understand videos everything is just superb so explanatory and easy to understand
Evaluation	A wonderful contribution to the profession. Congratulations on improving thousands of lives around the world with the generous sharing of this information. I have been blown away with the number of people doing.
Evaluation	Wonderful course, very good information, well organised. I have learned lot.
Evaluation	Information included in that course are amazing
Evaluation	The course was amazing Very well structured and organised. Learn a lot.

*FB* Facebook, *Wk* week.

^a^See Appendix 4 for all English response to Q14 of the Evaluation (post-MOOC Evaluation).

### REACTION: How did the participants react to the MOOC?

#### Post-MOOC evaluation

2281 participants completed the post-MOOC Evaluation. 98% of respondents rated the MOOC as either “*good*” (30%) or “*very good*” (68%) on all questions except the two questions related to the usefulness of the FB discussions and additional readings (see Supplementary File 14). Most (70%) indicated that they had already used the knowledge and skills learnt on the MOOC to improve themselves with 26% indicating that they had used the knowledge and skills to teach others.

#### Text analysis

The results for the three text analyses reflecting participants’ reaction to the MOOC are as follows.

Participants’ overall impressions of the MOOC: Participants stated that they “*learnt* [something]” or “l*earnt a lot*” 1500 times. They used the words “*amazing*”, “*awesome*”, “*brilliant*”, “*fantastic*”, “*great*”, “*superb*” or “*wonderful*”, 452 times (see Table 3).

**Table 3:** REACTION: Participants’ overall impressions of the MOOC.

Learnt/learned a lot	1500
Informative/useful/beneficial/Helpful	1067
Thanks/thank you	595
Amazing/awesome/brilliant/	
fantastic/great/superb/wonderful	452
Interesting	364
Easy/Simple	275
Enjoyed	264
Interact[ive]	149
Everything was good/worked well	144
Excellent/very good	142
Appreciate/grateful	104
Love[d]	100
Comprehensive/thorough	76
[great/good] Opportunity	72

The number of times the following positive words were used by participants to describe the MOOC. These were obtained by searching for the following words on three discussion threads of the English Facebook Group and the post-MOOC Evaluation.

The topics that participants enjoyed or valued learning about: The eight topics that participants most enjoyed learning about were: www.physiotherapyexercises.com (free online software to prescribe exercises), different assessment tools, wheelchair skills, motor training, strength training, neurological classification of SCI, autonomic dysreflexia and upper limb function (see Supplementary File 15).

The aspects of the learning experience that participants valued and/or enjoyed: Participants particularly enjoyed the number and diversity of videos (476 comments), the case studies (156 comments), using www.physiotherapyexercises.com (174 comments), the availability of the course book (392 comments), the self assessments and quizzes (108 comments), the overall structure and format (709 comments) and the exposure/opportunity to engage with people from different countries and cultures (138 comments) (see Supplementary File 16).

### BEHAVIOUR: Will the participants change their clinical practice or teach others differently in response to the MOOC?

#### Text analysis

The two notable clinical skills participants wrote about were (i) using what they had learnt to improve the way they taught patients motor skills (ii) using www.physiotherapyexercises.com to create exercise programs for their patients (see Table 2 for examples of comments). In particular, they commented on learning how to break complex motor tasks into simpler motor tasks for training purposes. They also talked about their intentions to change other aspects of their clinical practice and the way they taught students and junior clinicians (see Supplementary File 17 for examples of comments).

## Discussion

This study uses qualitative and quantitative data to determine the impact of our MOOC designed to train physiotherapists in the management of people with SCI. It also explores the learners’ experiences. Specifically, we sought answers to four key questions categorised under the headings: reach, effectiveness, reaction and behaviour. The results of this study are important because they will help organisations like ISCoS make decisions about using their scarce teaching resources, money and effort to run these MOOCs in the future. They will also help improve the conduct of future MOOCs (both on this specific topic and other topics).

One of the great strengths of our MOOC was its *reach*. Nearly 26,000 people from 169 different countries registered for the MOOC. Many were from countries where there may be few opportunities for physiotherapists to learn about SCI. We conservatively estimate that approximately 6250 (ie., 25% of those who started the MOOC) worked through most of the content (this is based on the number of participants who were still actively engaged on FB during week 5 and the number of people who completed the post-MOOC Knowledge Assessment and Evalution). We looked at the characteristics (country, language and level of SCI experience) of those who completed the pre- and post-MOOC Knowledge Assessments compared to the characteristics of those that registered for the MOOC. In so far as completion of the post-MOOC Knowledge Assessment reflects ongoing engagement, there was no obvious differences to indicate that certain types of participants registered but then did or did not complete the pre- or post-MOOC Knowledge Assessments.

The clear benefit of our MOOC was our ability to teach such a large number of people. To do the same in a tradional class-room setting would require 125 3-day workshops in most countries around the world (assuming 50 people per workshop). This is logistically and economically impossible for organisations like ISCoS to manage particularly if countries do not have their own teachers. Even in countries that do have physiotherapists with teaching and clinical expertise in SCI, there are still many barriers preventing participants from travelling to a central place for a three day face-to-face workshop particularly in geographically large countries such as India, China, Australia and Brazil (four of the the top five countries with the most participants and four of the top ten countries in the world with the largest land masses). And many people can not devote three days (plus travel) to one specific topic [16].

There may be an indirect reach of our MOOC which is difficult to measure and gauge. For example, for every person who registered for the MOOC, there may have been another 20 people who saw the advertisements for the MOOC. So potentially 0.5 million people were at least made aware of the possible need to learn about SCI. They may have also been exposed to ISCoS and the learning opportunities provided by ISCoS for the first time. This indirect reach contributes to raising the awareness of SCI in all corners of the world and may help to prioritise teaching of this topic.

The *effectiveness* of our MOOC is in part reflected in the change of scores on the Knowledge Assessments. This is, however, a problematic way of measuring effectiveness and knowledge because only 4016 of the 25,737 participants completed both the pre- and post-MOOC Knowledge Assessments, and this sample may not be reflective of all (for example, it may be a biased sample if those that had learnt the most were more likely to do the Knowledge Assessments). In addition, the usefulness of the Knowledge Assessments is limited because the questions need to be conducive to the multiple-choice format. So whilst some of the questions did require clinical reasoning, most merely required participants to recall facts. It did not measure changes in clinical skills. There were, however, many unsolicited comments made by participants that indicated they had learnt new things. There were also comments about increases in confidence. Perhaps in the future specific questions could be added to the post-MOOC Evaulation to better quantify changes in confidence.

Participants’ *reaction* to the MOOC is important to capture because it helps us understand what they did and did not enjoy about the learning experience, and how they will or will not use knowledge and skills learnt from the MOOC to improve themselves or to teach others more effectively. The post-MOOC Evaluations were overwhelmingly positive although some participants did not enjoy the FB discussions, a few wanted live streamed lectures and others had problems viewing the videos or did not enjoy the additional readings (which were optional). Of course, these findings may be vulnerable to response bias because possibly only those who enjoyed the MOOC completed the post-MOOC Evaluation [23]. The only way to overcome this potential bias is to ensure that either everyone who registered for the MOOC or a random sample of those that registered for the MOOC complete the post-MOOC Evaluation. We tried to encourage people to complete the post-MOOC Evaluation by stating that it needed to be completed in order to receive a Certificate of Completion. However, this was not enforced and many people were not interested in Certificates of Completion. In the future, we could perhaps consider paying a random sample of people to complete the post-MOOC Evaluations in order to better understand how all people perceived the MOOC. This could help us improve the MOOC. Nonetheless, we know that at least 2,235 people (98% of those who completed the post-MOOC Evaluation) were very satisfied with most aspects of the MOOC.

The two aspects of the MOOC that were less successful were the FB discussions and the additional readings. The FB discussions were included to create a sense of community because this is known to increase engagement with online courses. Many participants appeared to value the opportunity to hear from colleagues in all corners of the world. So this was a positive aspect of the FB groups. However, the FB discussion threads were also included to encourage participants to go away and read up about two or three particular topics each week, and to come online and together discuss what they had learnt. For some, this worked very well. However, clearly others were hoping for more than what a forum like this with so many people could ever achieve. Perhaps in future small breakaway FB groups could be created where participants could discuss the topics. This would, however, be very resource intensive. The best solution is probably to try and encourage people in the same locations or work places to come together and discuss and learn as a group. This would, however, be difficult to coordinate centrally.

It was interesting to see how many people enjoyed learning about www.physiotherapyexercises.com (free online software for prescribing exercises). They also valued the format of www.elearnSCI.org and, in particular, the use of so many short videos embedded in the content of real people with SCI and real SCI physiotherapists treating patients.

Of course, the best measure of the success of a MOOC like ours is whether it changes *behaviour* either with respect to how physiotherapists treat patients or how they teach others. And not surprisingly, this is very difficult to measure. People articulated their intentions to change practice and use what they had learnt to teach others but we do not know if they did as they intended, nor how many people felt the same way. It is difficult to provide a suggestion of how this important aspect of our MOOC could be objectively quantified in the future.

There are many possible factors that prevent people from fully engaging with MOOCs. It might be that a MOOC does not meet a person’s immediate educational or professional needs, or the content is not pitched at the right level (i.e., too easy or difficult). Often practical issues can play a role. For example, some participants may drop out of MOOCs because of difficulties navigating and using technology, or because of poor internet connection [16]. It may also be that some participants fail to maintain motivation because of the reduced opportunity to interact with their teachers and peers: an important source of social engagement and opportunity to have questions answered. Participants of MOOCs may also have a reduced commitment and/or sense of accountability because they are not answerable to a teacher or peers, and they have not paid anything to learn [18]. Consequently, participants may cease to engage in the face of any minor barrier that may otherwise be tolerated in a traditional classroom setting. Nonetheless, and despite the many barriers to MOOCs, they clearly appeal to many. And people come to MOOCs with different expectations, needs and commitments [7]. They are non-binding and as such it should not be surprising if people drop in and drop out [7].

In all, most of the results presented in this study point to the value and usefulness of our MOOC. It helped to increase physiotherapists’ knowledge, skills and confidence to manage people with SCI as well as to teach their students and patients. ISCoS and other professional organisations should consider developing similar MOOC for other professionals and on other topics to help upskill clinicians around the world in the management of people with SCI. However, careful consideration needs to be given to the design of MOOCs to ensure they adhere to the key adult teaching and learning principles identified as central to the success of any MOOC [21]. It is also important that effort is directed at devising ways to assess the impact of MOOCs on clinical practice.

### Supplementary information

Checklist

Supplementary File 1

Supplementary File 2

Supplementary File 3

Supplementary File 4

Supplementary File 5

Supplementary File 6

Supplementary File 7

Supplementary File 8

Supplementary File 9

Supplementary File 10

Supplementary File 11

Supplementary File 12

Supplementary File 13

Supplementary File 14

Supplementary File 15

Supplementary File 16

Supplementary File 17

## Data Availability

Most raw data are provided in the Supplementary Files. The authors will consider any reasonable requests for additional data.
