# Supplementary material for: The 2022 Massive Open Online Course (MOOC) to train physiotherapists in the management of people with spinal cord injuries: a qualitative and quantitative analysis of learners’ experiences and its impact
Source: Spinal Cord. 2023 Aug 14;61(11):615–23. doi: 10.1038/s41393-023-00922-1 (PMC10645583; doi:10.1038/s41393-023-00922-1)
Supplement: Supplementary file 2 — Supplementary File 1 [file 41393_2023_922_MOESM2_ESM.pdf]

Supplementary File 1: BACKGROUND: The topics, aims and objectives of each learning task.
There were between 2 and 4 learning tasks per week

Topic	Aim<br>**The aim of this task is to:**	Objectives<br>**At the end of this task participants will be able to:**
The Health Essentials	• Provide the background information that is essential for managing a person with SCI. This task uses simple case scenarios to illustrate important impairments and health conditions associated with SCI. Detailed information about these topics can be found in the e-learn doctors and nursing modules.	• Define the difference between a complete and incomplete SCI<br>• Describe common health conditions and impairments associated with SCI and their implications for physiotherapy<br>• List the key ways people with SCI manage their bladder and bowels and implications for physiotherapy
Principles of Management	• Provide a framework to help students and inexperienced physiotherapists formulate a physiotherapy program for people with SCI. Participants will learn a five-step process based on a problem-solving approach. The five steps are:<br>• Step 1: Assessing impairments, activities and participation<br>• Step 2: Setting goals which are specific, measurable, attainable, realistic and time bound (SMART)<br>• Step 3: Identifying key impairments<br>• Step 4: Identifying and administering treatments<br>• Step 5: Measuring outcomes	• Define the general principles of physiotherapy management<br>• Use ICF terminology to formulate a physiotherapy management plan<br>• Identify key impairments that are amenable to physiotherapy treatment
Assessment	• Provide an overview of a physiotherapy assessment for a person with SCI. Participants will learn the different aspects of a comprehensive assessment and the different types of assessments commonly used by physiotherapists in SCI.	• Explain the importance of a comprehensive assessment<br>• Identify some common ways of assessing impairments, activity limitations and participation restrictions<br>• Describe the steps involved in performing an assessment

Topic	Aim<br>**The aim of this task is to:**	Objectives<br>**At the end of this task participants will be able to:**
Setting Goals	• Provide an overview of the goal setting process for people with motor complete SCI. Participants will learn about the key muscles innervated at each level of injury and how this impacts on physical functioning.	• Identify the key muscles innervated at different levels of SCI<br>• Define the difference between C5 and C6 tetraplegia<br>• Set short and long term goals for people with complete SCI which are specific, measurable, attainable, realistic, time bound (SMART) and important to the patient<br>• List some of the key variables affecting outcome<br>• Describe the likely level of independence and mobility of people with different levels of SCI
Upper Limb Function	• Provide an overview of the implications of different patterns of upper limb paralysis on upper limb function. Participants will learn about the hand and upper limb function of people with different levels of tetraplegia.	• Describe the hand and upper limb function of people with motor complete C4 to C8 tetraplegia<br>• List the key determinants of a tenodesis grip<br>• Identify common impairments preventing learning of upper limb motor tasks<br>• Name some of the common measures of hand function
Wheelchair Mobility	• Provide an overview of how people with SCI mobilise in a manual and power wheelchair.	• Identify the key features of wheelchairs<br>• Describe how wheelchair set up can influence posture and function<br>• List some of the skills essential for mobilising in a power and manual wheelchair<br>• Understand the mechanics of performing a wheel stand<br>• Apply the principles of motor learning when teaching patients wheelchair skills

Topic	Aim<br>**The aim of this task is to:**	Objectives<br>**At the end of this task participants will be able to:**
Bed Mobility and Transfers: Understanding Movement	• Equip students and inexperienced clinicians with the skills to teach transfers and bed mobility to people with paraplegia and C6 tetraplegia. Emphasis will be on identifying the movement strategies commonly used by people with SCI to perform these motor tasks and the common impairments that contribute to limitations in function. The task involves viewing and analysing videos of skilled and novice performances of bed mobility and transfers.	• List the movement strategies used by people with C6 tetraplegia to roll, move from lying to sitting and transfer onto a bed<br>• List the movement strategies used by people with paraplegia to roll, move from lying to sitting, transfer onto a bed and transfer off the floor<br>• Analyse the movement strategies of skilled performances<br>• Identify common impairments that prevent learning of these motor tasks<br>• Develop a treatment plan based on impairments and activity limitations<br>• List the equipment that is useful for bed mobility and transfers<br>• Evaluate the success of treatments
Gait: Understanding Movement	• Provide an overview of the implications of different patterns of lower limb paralysis on gait. Participants will learn about the standing and walking options for people with common types of SCI.	• Identify the walking options for people with different types of SCI<br>• Identify the standing options and equipment required for people with different types of SCI<br>• Define the role of major lower limb muscles in gait<br>• List the implications of paralysis of major lower limb muscles on gait<br>• Describe the biomechanical principles of different types of orthoses<br>• Outline the principles of gait training

Topic	Aim<br>**The aim of this task is to:**	Objectives<br>**At the end of this task participants will be able to:**
Strength	• Provide an overview of the assessment and treatment of poor strength in people with SCI. Participants will learn about common examples of where strength imposes activity limitations and participation restrictions.	• List some common ways to assess strength<br>• Identify some examples of where weakness imposes activity limitations and participation restrictions<br>• List the key principles important for effective strength training<br>• Outline an appropriate program for increasing strength in people with tetraplegia and paraplegia
Contracture	• Provide an overview of the assessment and treatment of contractures for people with SCI. Participants will learn about common examples of where contractures impose activity limitations and participation restrictions.	• List some common ways to assess contractures<br>• Identify some examples of where contractures impose activity limitations and participation restrictions<br>• List the key principles important for effective contracture management<br>• Outline an appropriate program for treating and preventing contractures
Motor Skills	• Provide an overview of the assessment and treatment of motor skills in people with SCI. Participants will learn about common examples of where lack of skill results in activity limitations and participation restrictions.	• List some common ways to assess motor skills<br>• Identify some examples of where people with SCI need to learn new motor skills<br>• Identify some examples of where poor skill imposes activity limitations and participation restrictions<br>• List the key principles important for effective training of motor skills<br>• Outline an appropriate program for improving motor skills

**Topic**	**Aim**<br>**The aim of this task is to:**	**Objectives**<br>**At the end of this task participants will be able to:**
Fitness	• Provide an overview of the assessment and treatment of poor fitness in people with SCI. Participants will learn about common examples of where poor fitness imposes activity limitations and participant restrictions.	• List some common ways to assess fitness<br>• Identify some examples of where poor fitness imposes activity limitations and participation restrictions<br>• List the key principles important for improving fitness<br>• Outline an appropriate program for improving and maintaining fitness<br>• Understand the differences between training fitness in a person with high paraplegia versus a person with low paraplegia
Respiratory	• Provide an overview of respiratory function in people with SCI. Participants will learn about the common causes of poor respiratory function and key aspects of physiotherapy management.	• List the key features of a respiratory assessment<br>• Outline how SCI affects respiratory function<br>• Identify those at increased risk of respiratory complications<br>• Identify physiotherapy techniques appropriate for people with SCI<br>• Outline an appropriate program for improving and maintaining respiratory function
Pain	• Provide an overview of the assessment and treatment of pain in people with SCI. Participants will learn about the common causes of pain and key aspects of physiotherapy management.	• List some common ways to assess pain<br>• Identify some examples of where pain imposes activity limitations and participation restrictions<br>• List the key principles important for effective pain management<br>• Understand the difference between acute and chronic pain<br>• Understand the difference between nociceptive and neurological pain<br>• Outline an appropriate physiotherapy program for treating and preventing pain
