# Supplementary material for: The 2022 Massive Open Online Course (MOOC) to train physiotherapists in the management of people with spinal cord injuries: a qualitative and quantitative analysis of learners’ experiences and its impact
Source: Spinal Cord. 2023 Aug 14;61(11):615–23. doi: 10.1038/s41393-023-00922-1 (PMC10645583; doi:10.1038/s41393-023-00922-1)
Supplement: Supplementary file 3 — Supplementary File 2 [file 41393_2023_922_MOESM3_ESM.pdf]

Supplementary File 2: REACH: The number of registrants for each language

English	19,940 (77%)
Chinese	2,884 (11%)
Spanish	1,181 (5%)
Portuguese	1,073 (4%)
French	659 (3%)
**TOTAL**	**25,737**

[NB. 168 people registered for more than one language. They were only counted once in their non-English language)
