# Supplementary material for: The 2022 Massive Open Online Course (MOOC) to train physiotherapists in the management of people with spinal cord injuries: a qualitative and quantitative analysis of learners’ experiences and its impact
Source: Spinal Cord. 2023 Aug 14;61(11):615–23. doi: 10.1038/s41393-023-00922-1 (PMC10645583; doi:10.1038/s41393-023-00922-1)
Supplement: Supplementary file 4 — Supplementary File 3 [file 41393_2023_922_MOESM4_ESM.pdf]

Supplementary File 3: REACH: The countries of MOOC participants (as registered with each language)

Country	English	Chinese	French	Portuguese	Spanish	Total
Afghanistan	72	1	0	0	0	73
Albania	9	0	0	0	0	9
Algeria	11	0	9	0	0	20
American Samoa	2	2	0	0	0	4
Andorra	1	0	0	0	0	1
Angola	1	0	0	11	1	13
Argentina	13	0	0	0	176	189
Aruba	3	0	0	0	0	3
Australia	1,382	10	0	0	1	1,393
Austria	15	0	1	0	0	16
Azerbaijan	2	0	0	0	0	2
Bahamas	3	0	0	0	0	3
Bahrain	12	1	0	1	0	14
Bangladesh	67	1	0	0	0	68
Barbados	5	0	0	0	0	5
Belgium	44	1	9	0	0	54
Belize	1	0	0	1	1	3
Benin	0	0	35	0	0	35
Bermuda	5	0	0	0	0	5
Bhutan	43	0	0	0	0	43
Bolivia	2	0	0	0	5	7
Bosnia and Herzegovina	1	0	0	0	0	1
Botswana	23	0	0	0	0	23
Brazil	40	0	0	969	0	1,009
Brunei Darussalam	7	0	0	0	0	7
Bulgaria	10	0	0	0	0	10
Burundi	0	0	2	0	0	2
Cambodia	1	0	0	0	0	1
Cameroon	22	0	20	0	0	42
Canada	228	1	6	0	0	235
Cape Verde	0	0	0	1	0	1
Catalonia	0	0	0	0	1	1
Chad	1	1	1	0	0	3
Chile	11	3	0	0	288	302
China	31	2,750	0	1	0	2,782
Christmas Island	0	1	0	0	0	1
Colombia	15	0	0	0	76	91
Congo	1	0	35	0	0	36
Cook Islands	1	0	0	0	0	1

Country	English	Chinese	French	Portuguese	Spanish	Total
Costa Rica	15	0	1	0	68	84
Cote d'Ivoir	1	0	20	0	0	21
Croatia	3	0	0	0	0	3
Cuba	1	0	0	0	1	2
Cyprus	22	0	0	0	0	22
Czech Republic	2	0	0	0	0	2
Denmark	23	0	0	0	0	23
Djibouti	0	0	1	0	0	1
Dominica	1	0	0	0	0	1
Dominican Republic	3	0	0	0	6	9
Ecuador	1	0	0	0	19	20
Egypt	7,255	0	0	0	0	7,255
El Salvador	1	0	0	0	0	1
England	79	0	0	0	0	79
Eritrea	1	0	0	0	0	1
Estonia	5	0	0	0	0	5
Ethiopia	5	0	0	0	0	5
European Union	0	0	0	0	2	2
Fiji	8	0	0	0	0	8
Finland	19	0	0	0	0	19
France	20	0	396	5	3	424
French Polynesia	0	0	1	0	0	1
French Southern Territories	0	0	1	0	0	1
Gabon	0	0	3	0	0	3
Georgia	1	0	0	0	0	1
Germany	12	0	0	0	0	12
Ghana	43	1	0	0	0	44
Greece	169	0	0	0	0	169
Grenada	4	0	0	0	0	4
Guatemala	0	0	0	0	24	24
Guinea	0	0	1	0	0	1
Guyana	23	0	0	0	0	23
Haiti	2	0	15	0	0	17
Hungary	18	0	0	0	0	18
Iceland	2	0	0	0	0	2
India	2,579	0	1	0	0	2,580
Indonesia	25	0	0	0	0	25
Iran	4	0	0	0	0	4
Iraq	38	0	0	1	0	39
Ireland	89	0	0	0	0	89
Israel	58	0	1	0	0	59
Italy	38	0	1	2	2	43
Jamaica	28	0	0	0	0	28

Country	English	Chinese	French	Portuguese	Spanish	Total
Japan	8	0	0	0	0	8
Jordan	401	0	0	0	0	401
Kenya	34	0	0	0	0	34
Korea	5	0	0	0	0	5
Kuwait	66	0	0	0	0	66
Latvia	9	0	0	0	0	9
Lebanon	55	0	12	0	0	67
Libya	29	0	0	0	0	29
Lithuania	5	0	0	0	0	5
Macedonia	2	0	0	0	0	2
Madagascar	1	0	1	0	0	2
Malawi	35	0	0	0	0	35
Malaysia	270	7	0	0	0	277
Maldives	25	0	0	0	0	25
Mali	1	0	3	0	0	4
Malta	12	0	0	0	0	12
Mauritania	1	0	0	0	0	1
Mauritius	5	0	0	0	0	5
Mexico	38	0	0	0	273	311
Moldova	1	0	0	0	0	1
Mongolia	6	0	0	0	0	6
Montenegro	5	0	0	0	0	5
Morocco	17	0	26	0	0	43
Mozambique	0	0	0	6	0	6
Myanmar	46	0	0	0	0	46
Namibia	32	0	0	0	0	32
Nepal	137	0	0	0	0	137
Netherlands	24	0	0	0	0	24
Netherlands Antilles	5	0	0	0	0	5
New Zealand	186	0	0	0	0	186
Nicaragua	0	0	0	0	4	4
Niger	0	0	9	0	0	9
Nigeria	630	0	0	0	0	630
Norway	28	0	0	0	0	28
Oman	44	0	0	0	0	44
Other	72	100	1	0	0	173
Pakistan	568	0	0	0	0	568
Palestinian Territory	180	0	0	0	0	180
Panama	1	0	0	0	0	1
Papua New Guinea	1	0	0	0	0	1
Paraguay	0	0	0	0	1	1
Peru	13	0	0	0	97	110

Country	English	Chinese	French	Portuguese	Spanish	Total
Philippines	176	0	0	0	0	176
Poland	17	0	0	0	0	17
Portugal	7	0	0	73	0	80
Puerto Rico	12	0	0	0	4	16
Qatar	72	0	4	0	1	77
Reunion	0	0	6	0	0	6
Romania	111	0	1	0	1	113
Russian Federation	8	0	0	0	0	8
Rwanda	34	0	1	0	0	35
Samoa	1	0	0	0	0	1
Saudi Arabia	655	0	0	0	0	655
Scotland	5	0	0	0	0	5
Serbia	2	0	0	0	0	2
Singapore	187	1	0	0	0	188
Slovenia	5	0	0	0	0	5
Solomon Islands	3	0	0	0	0	3
Somalia	25	0	0	0	0	25
South Africa	338	0	0	0	0	338
Spain	13	0	1	0	99	113
Sri Lanka	96	0	0	0	0	96
Sudan	399	0	0	0	0	399
Suriname	5	0	0	0	0	5
Swaziland	1	0	0	0	0	1
Sweden	18	0	0	0	0	18
Switzerland	18	0	6	0	0	24
Syrian Arab Republic	40	0	0	0	0	40
Tanzania	39	0	0	0	0	39
Thailand	19	0	0	0	0	19
Togo	1	0	16	0	0	17
Trinidad and Tobago	5	0	0	0	0	5
Tunisia	7	0	10	0	0	17
Turkey	296	0	1	0	0	297
Turks and Caicos Islands	3	0	0	0	0	3
Uganda	40	0	0	0	0	40
Ukraine	77	0	0	0	0	77
United Arab Emirates	152	0	0	1	0	153
United Kingdom	917	1	0	1	0	919
United States	230	1	0	0	0	231
Uruguay	1	0	0	0	21	22
Venezuela	1	0	0	0	6	7

Country	English	Chinese	French	Portuguese	Spanish	Total
Vietnam	70	1	1	0	0	72
Wales	6	0	0	0	0	6
Yemen	52	0	0	0	0	52
Zambia	92	0	0	0	0	92
Zimbabwe	3	0	0	0	0	3
Total	19,940	2,884	659	1,073	1,181	25,737
