# Supplementary material for: The 2022 Massive Open Online Course (MOOC) to train physiotherapists in the management of people with spinal cord injuries: a qualitative and quantitative analysis of learners’ experiences and its impact
Source: Spinal Cord. 2023 Aug 14;61(11):615–23. doi: 10.1038/s41393-023-00922-1 (PMC10645583; doi:10.1038/s41393-023-00922-1)
Supplement: Supplementary file 5 — Supplementary File 4 [file 41393_2023_922_MOESM5_ESM.pdf]

Supplementary File 4: REACH: The professional experience of the participants.

Details shown according to the language that they registered on the MOOC.

Experience	English	Chinese	French	Portuguese	Spanish	Total
Undergraduate Student	4,775	663	238	426	253	6,355
Postgraduate Student	5,358	164	53	218	99	5,892
< 1 year SCI Experience	4,235	1,025	172	158	379	5,969
2 - 5 years SCI Experience	3,012	620	112	109	240	4,093
> 5 years SCI Experience	2,560	412	84	162	210	3,428
