# Supplementary material for: The 2022 Massive Open Online Course (MOOC) to train physiotherapists in the management of people with spinal cord injuries: a qualitative and quantitative analysis of learners’ experiences and its impact
Source: Spinal Cord. 2023 Aug 14;61(11):615–23. doi: 10.1038/s41393-023-00922-1 (PMC10645583; doi:10.1038/s41393-023-00922-1)
Supplement: Supplementary file 6 — Supplementary File 5 [file 41393_2023_922_MOESM6_ESM.pdf]

## Supplementary File 5: REACH: The number of participants who completed the post-MOOC Evaluation

| | |
|---|---|
| English | 1,571 |
| Spanish | 176 |
| Portuguese | 74 |
| French | 186 |
| Chinese | 274 |
| **TOTAL** | **2,281** |
