# Supplementary material for: The 2022 Massive Open Online Course (MOOC) to train physiotherapists in the management of people with spinal cord injuries: a qualitative and quantitative analysis of learners’ experiences and its impact
Source: Spinal Cord. 2023 Aug 14;61(11):615–23. doi: 10.1038/s41393-023-00922-1 (PMC10645583; doi:10.1038/s41393-023-00922-1)
Supplement: Supplementary file 7 — Supplementary File 6 [file 41393_2023_922_MOESM7_ESM.pdf]

Supplementary File 6: REACH: The number of people who joined each Facebook or Weibo group and the number of comments and reactions

Each Facebook and Weibo group had between 10 and 30 threads that were posted by the MOOC Co-ordinators. Participants could only post comments to each thread for one week before the threads were closed. Participants could not initiate new threads.

	No. who joined	Active members	Comments	Reactions
English	13,600	12,228	11,679	17,249
Spanish	847	706	1,530	2,612
Portuguese	457	349	923	979
French	490	442	1,428	617
Chinese	904	-	2365	724
**TOTAL**	16,298	13,725	17,925	22,181

[NB. These stats were downloaded from Facebook on 14/12/22 for English, Spanish, French and Portuguese. The statistics for the Weibo Chat were provided by the Chinese language MOOC Co-ordinator.]
