# Supplementary material for: The 2022 Massive Open Online Course (MOOC) to train physiotherapists in the management of people with spinal cord injuries: a qualitative and quantitative analysis of learners’ experiences and its impact
Source: Spinal Cord. 2023 Aug 14;61(11):615–23. doi: 10.1038/s41393-023-00922-1 (PMC10645583; doi:10.1038/s41393-023-00922-1)
Supplement: Supplementary file 8 — Supplementary File 7 [file 41393_2023_922_MOESM8_ESM.pdf]

Supplementary File 7: REACH: Number of posts to the Facebook threads each week (English only)

Each discussion thread was only open for one week and the numbers do not include those posting to the 4 other languages.

	**No. of posts**
Prior to Week 1 - Introduction	1.4K
Week 1 - Discussion thread #2 - Pressure ulcers	1.2K
Week 1 - Discussion thread #1 - Classification of SCI	1K
Week 2 - Discussion thread #1 - Assessment	729
Week 2 - Discussion thread #2 – Wheelchair skills	679
Week 2 - Discussion thread #2 – What have your learnt	821
Week 3 - Discussion thread #2 - Gait	770
Week 3 - Discussion thread #1 - Transfers	652
Week 3: Post a photo of your study group	322
Week 4 - Discussion thread #1 - Passive movements and stretch	500
Week 4 - Discussion thread #2 – Create an exercise program	872
Week 5 - Discussion thread #1 - Advice to preserve the shoulders	308
Week 5 - Discussion thread #2 - Staying fit and healthy	381
Week 5 - Discussion thread 3 - Summary of MOOC	639
**TOTAL**	10,273
