# Supplementary material for: The 2022 Massive Open Online Course (MOOC) to train physiotherapists in the management of people with spinal cord injuries: a qualitative and quantitative analysis of learners’ experiences and its impact
Source: Spinal Cord. 2023 Aug 14;61(11):615–23. doi: 10.1038/s41393-023-00922-1 (PMC10645583; doi:10.1038/s41393-023-00922-1)
Supplement: Supplementary file 9 — Supplementary File 8 [file 41393_2023_922_MOESM9_ESM.pdf]

Supplementary File 8: REACH: Number of emails sent to participants each week compared with the number received and opened

(Email sent 2 weeks prior - Wednesday 26th Oct 2022 (as of 2/11/22)

	No. sent	No. received	Opens	Unsubscribed
English	10,324	10,075	6,873	5
Chinese	2,489	2,385	2,289	0
Spanish	721	716	788*	0
Portuguese	528	525	514	0
French	169	167	153	0
Overall	14,084	14,077	10,617*	5

* Some participants opened the email multiple times

Emails sent on beginning of week 1 - Sunday 6th Nov 2022 (as of 21/11/22)

	No. sent	No. received	Opens	Unsubscribed
English	16,995	16,609	18,094*	15
Chinese	2,620	2,583	4,469*	0
Spanish	1,089	1,084	761	1
Portuguese	1,031	1,023	961	0
French	436	424	729*	1
Overall	22,171	21,723	25,014*	17

* Some participants opened the email multiple times

Emails sent on beginning of week 2 - Sunday 13th Nov 2022 (as of 21/11/22)

	No. sent	No. received	Opens	Unsubscribed
English	18,930	18,615	15,168	19
Chinese	2,800	2,762	1,034	0
Spanish	1,158	1,151	717	1
Portuguese	1,073	1,064	725	1
French	613	607	416	2
Overall	24,574	24,199	18,060	23

* The content of the emails could be accessed on the MOOC website so it is possible that participants did not feel the need to read them after the first week.

Emails sent on beginning of week 3 - Sunday 20th Nov 2022 (as of 21/11/22)

	No. sent	No. received	Opens	Unsubscribed
English	19,456	19,095	6,583	14
Chinese	2,873	2,812	546	0
Spanish	1,227	1,215	509	2
Portuguese	1,092	1,087	238	2
French	654	651	265	1
Overall	25,302	24,860	8,141	19

* The content of the emails could be accessed on the MOOC website so it is possible that participants did not feel the need to read them after the first week.

Emails sent on beginning of week 4 - Sunday 27th Nov 2022 (as of 15/12/22)

	No. sent	No. received	Opens	Unsubscribed
English	19,342	19,065	8,195	14
Chinese	2,874	2,520	647	0
Spanish	1,229	1,222	662	0
Portuguese	1,087	1,081	384	2
French	659	654	358	1
Overall	25,191	24,542	10,246	17

* The content of the emails could be accessed on the MOOC website so it is possible that participants did not feel the need to read them after the first week.

Emails sent on beginning of week 5 - Sunday 4th Dec 2022 (as of 15/12/22)

	No. sent	No. received	Opens	Unsubscribed
English	19,426	19,157	7,947	11
Chinese	2,873	2,828	745	0
Spanish	1,229	1,218	625	0
Portuguese	1,085	1,079	365	0
French	658	654	634	1
Overall	25,271	24,936	10,316	12

* The content of the emails could be accessed on the MOOC website so it is possible that participants did not feel the need to read them after the first week.
