# Supplementary material for: The 2022 Massive Open Online Course (MOOC) to train physiotherapists in the management of people with spinal cord injuries: a qualitative and quantitative analysis of learners’ experiences and its impact
Source: Spinal Cord. 2023 Aug 14;61(11):615–23. doi: 10.1038/s41393-023-00922-1 (PMC10645583; doi:10.1038/s41393-023-00922-1)

Glinsky JV, Ilha I, Xiong Y, Gomez G, Rostagnor S, Martin Manjarrer S, Tranter K, Muldoon S, Weerts E and Harvey LA (2023) The 2022 Massive Open Online Course (MOOC) to train physiotherapists in the management of people with spinal cord injuries: a qualitative and quantitative analysis of learners' experiences and its impact. Spinal Cord

Supplementary File 9: REACH: The number of unique page views of the weekly instructions on the English **version of www.SCIMOOC.org**

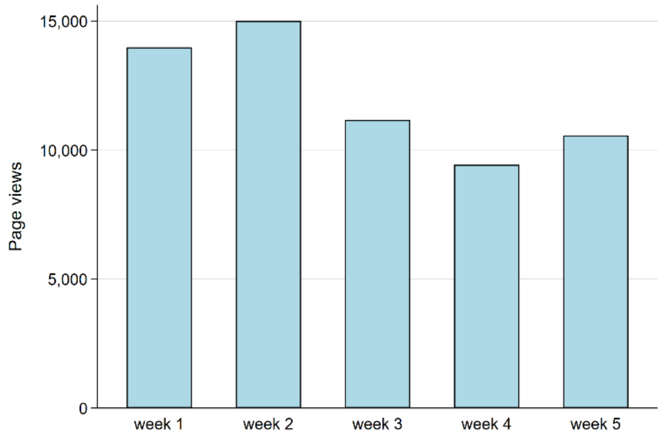

Supplement: Supplementary file 10 — Supplementary File 9 [file 41393_2023_922_MOESM10_ESM.pdf]
