# Supplementary material for: The 2022 Massive Open Online Course (MOOC) to train physiotherapists in the management of people with spinal cord injuries: a qualitative and quantitative analysis of learners’ experiences and its impact
Source: Spinal Cord. 2023 Aug 14;61(11):615–23. doi: 10.1038/s41393-023-00922-1 (PMC10645583; doi:10.1038/s41393-023-00922-1)
Supplement: Supplementary file 11 — Supplementary File 10 [file 41393_2023_922_MOESM11_ESM.pdf]

Supplementary File 10: REACH: Number of views and unique views of pages on the English version of www.SCIMOOC.org

	Page views	Unique views
Overview	7,015	5,113
To get started	8,392	5,533
Pre-MOOC Knowledge Assessment	12,793	3,739
**Week 1†**	22,978	13,985
**Week 2**	22,759	15,008
**Week 3**	15,903	11,171
**Week 4**	13,881	9,435
**Week 5**	14,278	10,568
Post-MOOC Knowledge Assessment	15,985	10,808
