# Supplementary material for: The 2022 Massive Open Online Course (MOOC) to train physiotherapists in the management of people with spinal cord injuries: a qualitative and quantitative analysis of learners’ experiences and its impact
Source: Spinal Cord. 2023 Aug 14;61(11):615–23. doi: 10.1038/s41393-023-00922-1 (PMC10645583; doi:10.1038/s41393-023-00922-1)
Supplement: Supplementary file 12 — Supplementary File 11 [file 41393_2023_922_MOESM12_ESM.pdf]

Supplementary File 11: Usage of www.physiotherapyexercises.com

Participants were required to create exercise booklets for a person with SCI during week 4 of the MOOC

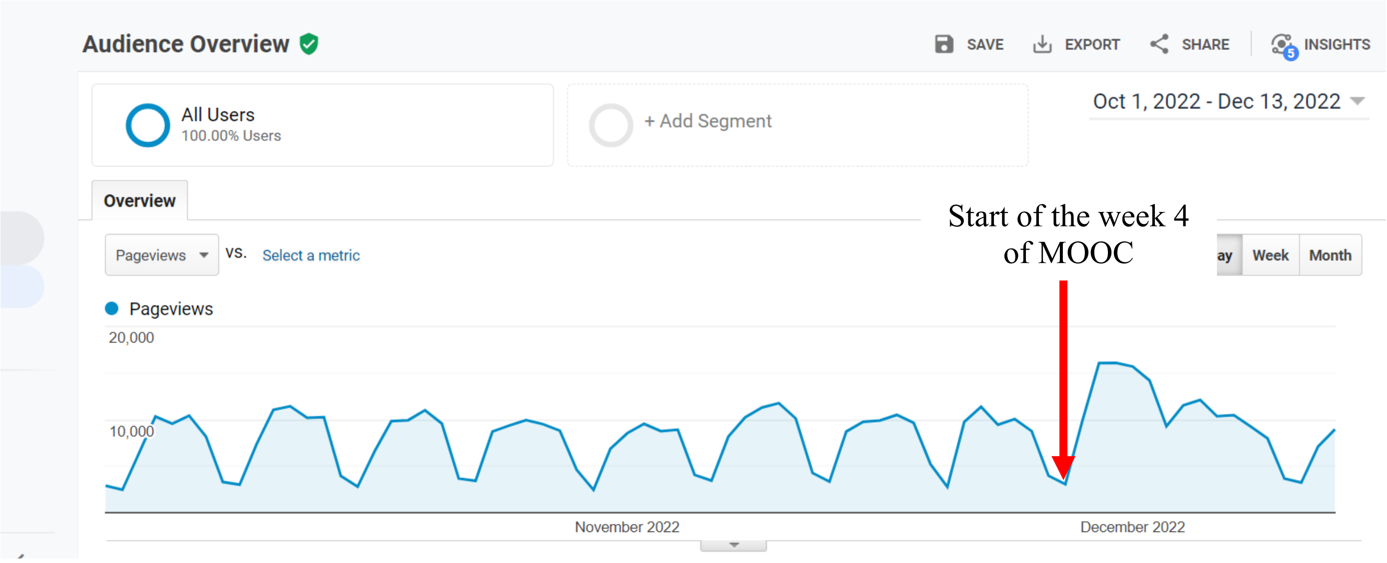

[The maximal number of views per day was 16,000].
