# Supplementary material for: The 2022 Massive Open Online Course (MOOC) to train physiotherapists in the management of people with spinal cord injuries: a qualitative and quantitative analysis of learners’ experiences and its impact
Source: Spinal Cord. 2023 Aug 14;61(11):615–23. doi: 10.1038/s41393-023-00922-1 (PMC10645583; doi:10.1038/s41393-023-00922-1)

Supplementary File 12: EFFECTIVENESS: Results of pre- and post-MOOC Knowledge Assessment

(all data are medians, interquartile ranges and top and bottom 5%)

All participants who did either (or both) the pre- and post-MOOC Knowledge Assessment (pre-MOOC, n = 10,206; post-MOOC, n = 4,873). Some participants had multiple attempts. The data reflects the results of their first attempt.

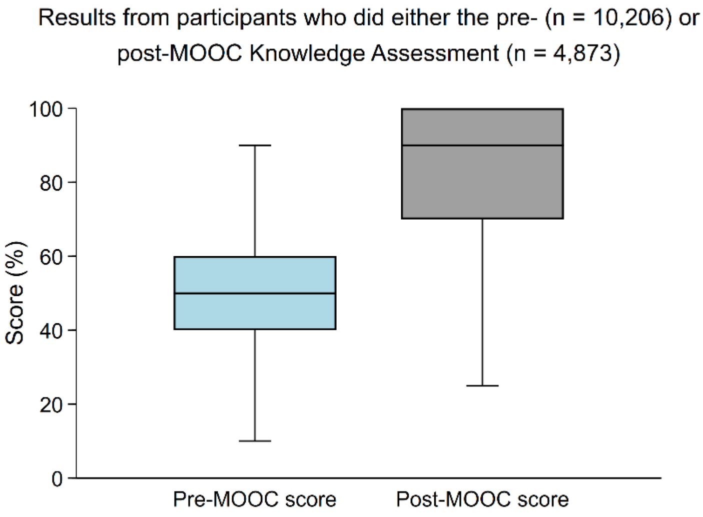

Supplement: Supplementary file 13 — Supplementary File 12 [file 41393_2023_922_MOESM13_ESM.pdf]
