# Supplementary material for: The 2022 Massive Open Online Course (MOOC) to train physiotherapists in the management of people with spinal cord injuries: a qualitative and quantitative analysis of learners’ experiences and its impact
Source: Spinal Cord. 2023 Aug 14;61(11):615–23. doi: 10.1038/s41393-023-00922-1 (PMC10645583; doi:10.1038/s41393-023-00922-1)
Supplement: Supplementary file 14 — Supplementary File 13 [file 41393_2023_922_MOESM14_ESM.pdf]

Supplementary File 13: EFFECTIVENESS: Examples of comments on Facebook the post-MOOC Evaluation indicative of a change in confidence

Source	Comment
FB, Wk 2	I feel more **confident** now when approaching a SCI patient and educating/training them about tenodesis grip
FB, Wk 2	This week has taught me a lot of techniques that are a little beyond the basics, this has given me **confidence** to go ahead and be part of that aspect of a patients rehab.
FB, Wk 2	I feel more **confident** teaching these skills to my patients now.
FB, Wk 2	I have also gained **confidence** in teaching wheelchair skills and knowing how to maximise safety whilst doing so, allowing the patients to also feel more confident when practicing.
FB, Wk 2	I loved learning about different strategies to improve a tenodesis grip….. it's been great to increase my knowledge and feel more **confident** in this area. Thank you!
FB, Wk 2	I have learned a lot this week. In particular something I had little experience of was how to complete wheelchair mobility on curbs/steps. I would feel more **confident** to teach patients to perform these skills now. Thank you.
FB, Wk 5	I feel so much more **confident** in treating those with spinal cord injuries.
FB, Wk 5	Now I'm more **confident** to work with patients with SCI.
FB, Wk 5	The course has been excellent. I've learned a lot of general background information that gives me more **confidence** in my wheelchair & seating work
FB, Wk 5	I feel a lot more **confident** to manage the few patients I come across in my rehab setting.
FB, Wk 5	now I feel more **confident** and get a a lot of insight and pool of knowledge from this course
FB, Wk 5	This course has helped boost my **confidence** in providing care as a Physiotherapist.
FB, Wk 5	…after receiving this course I feel much more **confident** about how to handle it.
Evaluation	The course was excellent. I feel more **confident** that I can help someone with SCI.
Evaluation	It helped me be more **confident** while treating SCI patients
Evaluation	Being a junior clinician, this course has given me **confident** to competently manage SCI.
Evaluation	I now feel alot more **confident**
Evaluation	I'm more **confident** to handle SCI patients. thank you.

Legend: FB: Facebook; Wk: week
