# Supplementary material for: The 2022 Massive Open Online Course (MOOC) to train physiotherapists in the management of people with spinal cord injuries: a qualitative and quantitative analysis of learners’ experiences and its impact
Source: Spinal Cord. 2023 Aug 14;61(11):615–23. doi: 10.1038/s41393-023-00922-1 (PMC10645583; doi:10.1038/s41393-023-00922-1)

Supplementary File 14: REACTION: Responses to each individual question of the post-MOOC Evaluation

(number of respondents = 2,281)

Q1. The course was enjoyable to work through

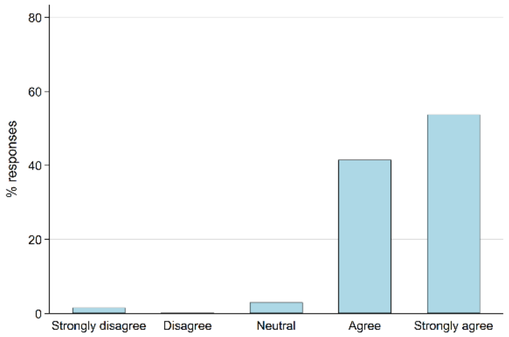

Q2. The course made good use of photographs, videos and illustrations

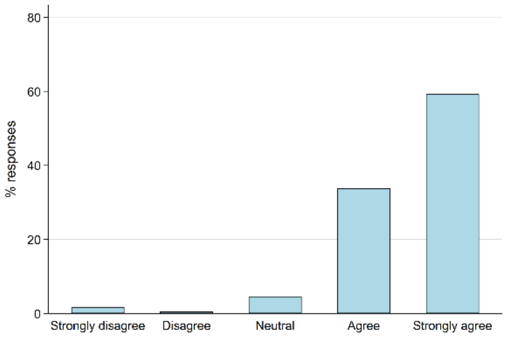

Q3. The course was an appropriate length (i.e., it was not too long)

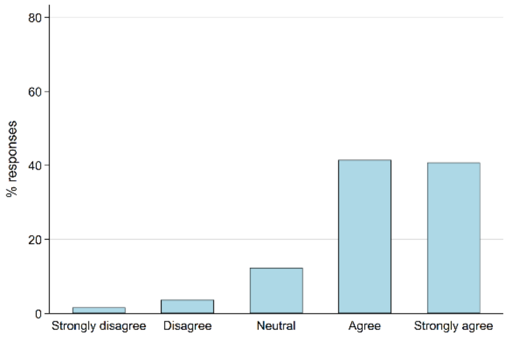

Q4. The course was interactive and required me to think and respond to questions

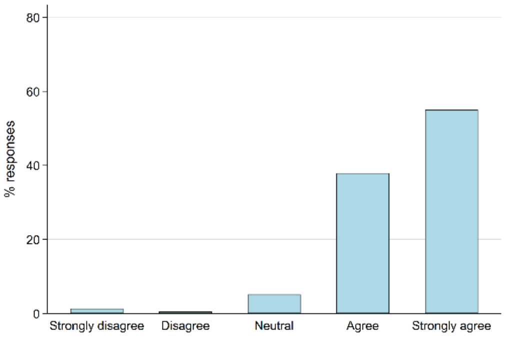

Q5. The course contained the information I needed

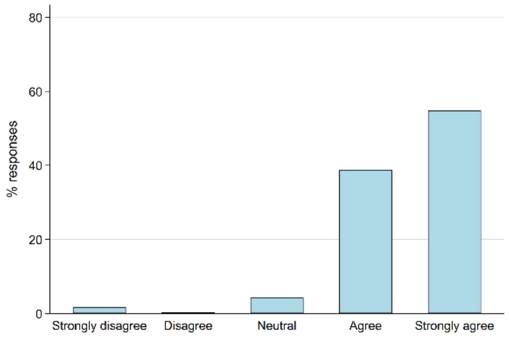

Q6. The course would be useful for students and junior clinicians (ie., not too hard)

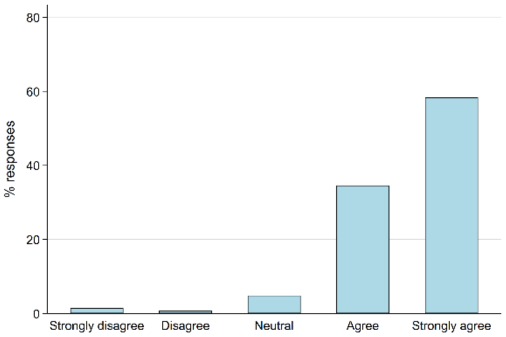

Q7. I would recommend this course to students and junior clinicians

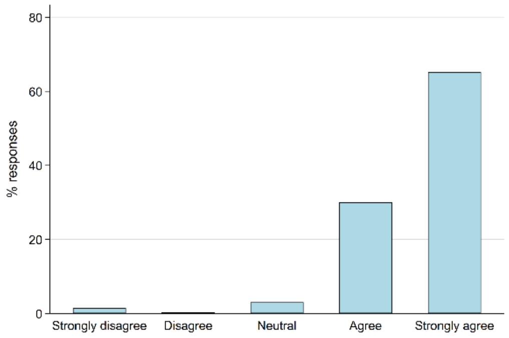

Q8. The course represented people from a variety of different cultures and countries

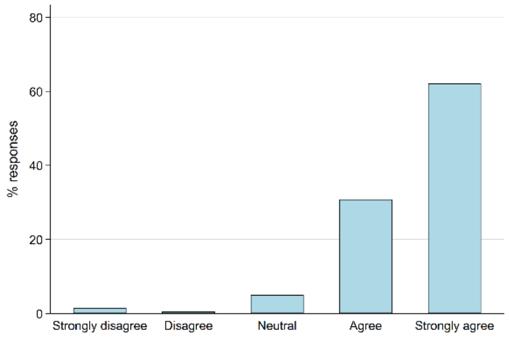

Q9. The course presented information in many different ways

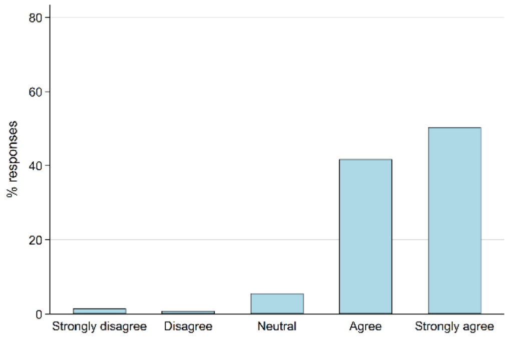

Q10. The self-assessments were helpful

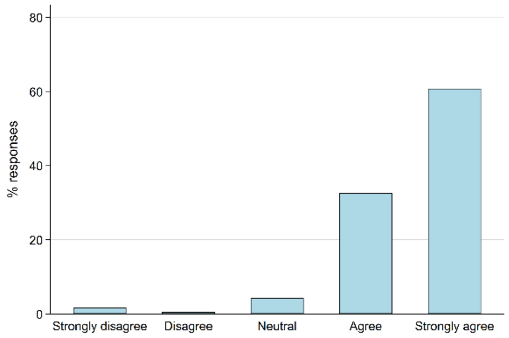

Q11. The Facebook discussion page was useful

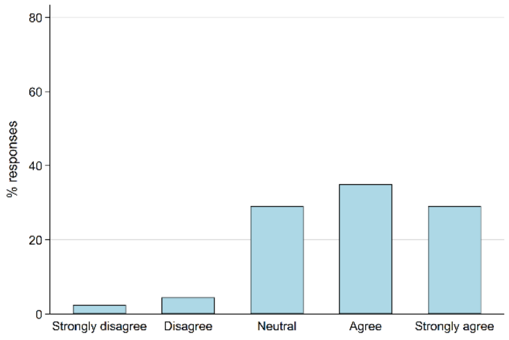

Q12. The additional readings were useful

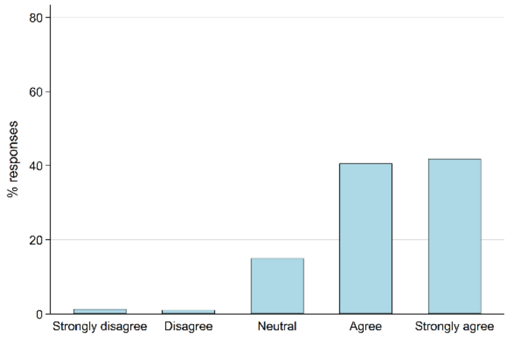

Q13. I have used the knowledge and skills I have learnt from the course to:

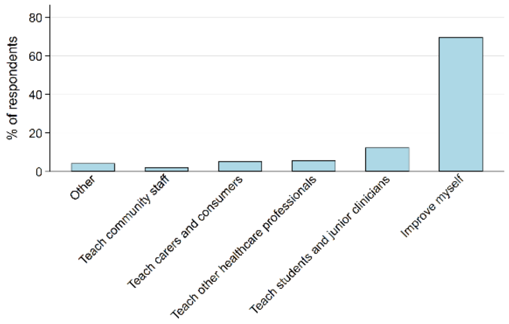

Q14. Overall rating of the course

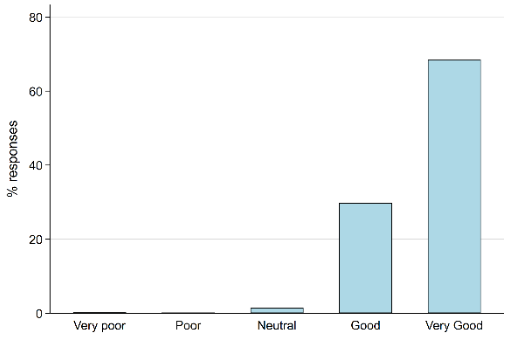

Supplement: Supplementary file 15 — Supplementary File 14 [file 41393_2023_922_MOESM15_ESM.pdf]
