# Supplementary material for: The 2022 Massive Open Online Course (MOOC) to train physiotherapists in the management of people with spinal cord injuries: a qualitative and quantitative analysis of learners’ experiences and its impact
Source: Spinal Cord. 2023 Aug 14;61(11):615–23. doi: 10.1038/s41393-023-00922-1 (PMC10645583; doi:10.1038/s41393-023-00922-1)
Supplement: Supplementary file 16 — Supplementary File 15 [file 41393_2023_922_MOESM16_ESM.pdf]

Supplementary File 15: REACTION: The topics that participants enjoyed or valued learning about

The number of times the following words were used by participants to describe what they had learnt or valued learning about. These were obtained by searching for the following words on three discussion threads of the English Facebook Group and the post-MOOC Evaluation.

Assessments/outcomes measures.... 571
Tenodesis/hand 309
Physiotherapyexercises.com 174
Autonomic dysreflexia.................... 148
Wheelchair skills............................. 123
Strength... 120
Sub-tasks/motor tasks 96
