# Supplementary material for: The 2022 Massive Open Online Course (MOOC) to train physiotherapists in the management of people with spinal cord injuries: a qualitative and quantitative analysis of learners’ experiences and its impact
Source: Spinal Cord. 2023 Aug 14;61(11):615–23. doi: 10.1038/s41393-023-00922-1 (PMC10645583; doi:10.1038/s41393-023-00922-1)
Supplement: Supplementary file 17 — Supplementary File 16 [file 41393_2023_922_MOESM17_ESM.pdf]

Supplementary File 16: REACTION: The aspects of the learning experience that participant valued or enjoyed

The number of times the following words were used by participants to describe aspects of the learning experience that they valued or enjoyed. These were obtained by searching for the following words on three discussion threads of the English Facebook group and the post-MOOC Evaluation.

Structure/format 709
The embedded videos 476
Availability of the course book....... 392
Using www.physiotherapyexercises.com 174
Case [studies] 156
Exposure to, and opportunity to engage with, people from different countries/cultures 138
Self assessments/quizzes................. 108
