# Supplementary material for: The 2022 Massive Open Online Course (MOOC) to train physiotherapists in the management of people with spinal cord injuries: a qualitative and quantitative analysis of learners’ experiences and its impact
Source: Spinal Cord. 2023 Aug 14;61(11):615–23. doi: 10.1038/s41393-023-00922-1 (PMC10645583; doi:10.1038/s41393-023-00922-1)
Supplement: Supplementary file 18 — Supplementary File 17 [file 41393_2023_922_MOESM18_ESM.pdf]

Supplementary File 17: BEHAVIOUR: Examples of comments on Facebook and the post-MOOC Evaluation indicative of an intention to change what or how participants teach other clinicians or students

Source	Comment
FB, Wk 2	I really like how the educational material is presented. I am a teacher and this is a very important lesson for me.
FB, Wk 2	What I've learned new that I intend to use is cramming down task training
FB, Wk 5	I enjoyed the activities from now on i will use this way of teaching it's more exciting than just giving new ideas the old way
Evaluation	i will also use to **improve my teaching** of students and junior staff
Evaluation	I plan to use some of the resources from this course **with my students**
Evaluation	Helpful to … **teach** my students and collegues
Evaluation	I also plan to **present to the staff** at my hospital on what i have learned from the course.
Evaluation	I will not only improve myself but will **share the knowledge** I gained through this course to improve the SCI care givers, students, SCI patients and physio colleague that will ultimately improve the quality of SCI care
Evaluation	**I will use** the knowledge and skills to …teach more junior staff and other healthcare professionals, patients, families and carers
Evaluation	**I will definitely use what I have learned to …..** help others, including my colleagues, students, and other healthcare professionals, improve as well!
Evaluation	It will help … **improve my teaching skills** for the SCI unit in the PTA program at a community college that I teach at.
Evaluation	it inspired me to go through the course for the overview and **to learn about how to teach student**
Evaluation	will also use to **improve my teaching of students and junior staff**

Legend: FB: Facebook; Wk: week
